# Comparison of Different Surgical Systems for Treatment of Early-onset Scoliosis in the Context of Release of Titanium Ions

**DOI:** 10.1097/BRS.0000000000003846

**Published:** 2020-12-07

**Authors:** Anna Danielewicz, Magdalena Wójciak, Jan Sawicki, Sławomir Dresler, Ireneusz Sowa, Michał Latalski

**Affiliations:** a Department of Paediatric Orthopaedics, Medical University of Lublin, Lublin, Poland; b Department of Analytical Chemistry, Medical University of Lublin, Lublin, Poland.

**Keywords:** child, early-onset scoliosis, growth friendly techniques, guided growth systems, ICP-OES, metal debris, spine, surgery, titanium, traditional growing rod, vertical expandable prosthesis titanium rib

## Abstract

Case–control study.

**Objective.:**

The aim of the study was to compare growth-friendly systems: traditional growing rod, guided growth systems (GGS), and vertical expandable prosthetic titanium rib in the context of titanium release.

**Summary of Background Data.:**

The problem of scoliosis affects even up to 3% of the population, and ca. 0.1% of patients need surgical treatment. Surgical treatment carries the risk of a long-term presence of implants in the organism, which may result in release of metal ions into the tissues and bloodstream.

**Methods.:**

Seventy-one patients (13.5 ± 3.54 years’ old) were treated for spinal deformity using various surgical systems and the samples of paraspinal tissue, blood, nails, and hair were collected before and after treatment. The quantification of titanium was performed using inductively coupled plasma optical emission spectrometry.

**Results.:**

The metallic particles were released into the peri-implant tissue, and the greatest amounts of titanium were detected in patients with GGS. The concentration of soluble titanium forms in subcutaneous tissue (ST) was low and do not statistically differ from control. The average titanium content in the paraspinal tissue in patients with GGS was two- to three-fold higher than the average value in the other investigated groups. A slightly increased level of titanium compared with the control was noted in all studied groups of patients. The highest content of titanium in blood was observed in patients with the GGS system.

**Conclusion.:**

Neverless the system used, the concentration of soluble titanium forms in both ST and blood was only slightly higher than in the control and did not exceed the allowable levels. The increased level of titanium with GGS system is probably associated with the friction between implant components, whereas the components in the other systems are immobile relative to each other.

**Level of Evidence:** 3

The problem of scoliosis affects even up to 2% to 3% of the population. It is defined as deformation of the spine >10° in the coronal plane. In addition to the lower quality of life due to pain and cosmetic aspects, scoliosis may lead to serious problems, for example, cardiorespiratory dysfunction and an increased risk of pulmonary problems.^1,2^ When the spinal deformity arises in patients younger than 10 years, it is called early-onset scoliosis (EOS). The etiology of EOS is diversified and includes five main types: idiopathic, congenital, thoracogenic, neuromuscular, and syndromic. However, regardless of the etiology of the curvature, EOS is a challenge for treatment because its goal is not only to correct the deformity, but also to slow down the progression and allow further growth of the chest and spine. These actions can prevent serious health consequences, including the inability of the thorax to support respiration and proper lung development referred to as thoracic insufficiency syndrome (TIS).^3^ Observation with rehabilitation or bracing to prevent progression of the spinal curvature are the modes of treatment in a majority of cases; however, ca 0.1% of patients need surgical treatment to correct the deformity and to ensure conditions for further proper growth of the chest and spine.^3^ Surgical treatment involves spinal instrumentation with the use of rods and screws made of metallic biomaterials mostly composed of titanium. Titanium is regarded as a relatively inert and biocompatible metal; however, it remains in the body for several years and can be gradually released into surrounding tissues over time as a result of corrosion and mechanical processes, for example, friction, bending, and scratching.^4^ It can also penetrate into the circulatory or lymphatic systems in the form of nanoparticles, ionic forms, organometallic complexes, or oxides^5,6^ and can thus be transferred to other tissues.^7,8^ Released titanium can exert adverse effects, for example, on bone formation, and initiate acute and chronic inflammation. It has been evidenced that titanium increases the level of macrophages and cytokines and stimulates the synthesis of prostaglandin and interleukin.^9,10^ The chirurgical treatment of EOS is an extremely difficult task. There are some useful surgical systems for correction of spinal deformities, and the choice of an appropriate one depends on the type and etiology of scoliosis as well as the age of the patient. One of the growth friendly systems, that is, the traditional growing rod (TGR), is applied most commonly but needs repetitive surgeries. TGR incorporates proximal and distal hooks or screw anchors on the deformed spine, joined by rods with connectors that allow serial distractions between the rods. Guided growth systems (GGS) allow spine growth with simultaneous correction of the deformation without the need for repetitive surgeries. In GGS, the spine is straightened with spinal implants that allow the vertebrae to grow along the path of the rods. As in the case of TGR, the vertical expandable prosthetic titanium rib (VEPTR) is used in patients who are up to 5 years’ old to prevent the development of TIS. The aim of present study was to compare these systems in the context of titanium release. The level of titanium in subcutaneous and paraspinal tissue as well as in blood, hair, and nails was assessed with the use of inductively coupled plasma optical emission spectrometry (ICP-OES). The study assessed whether: the concentration of titanium ions in tissues is significantly higher in children with spine instrumentation; the level of metal depends on the type of instrumentation; mechanical damage to the implant increases the concentration of metal ions in tissues.

## EXPERIMENTAL SECTION

### Patient Selection

**Figure 1:**
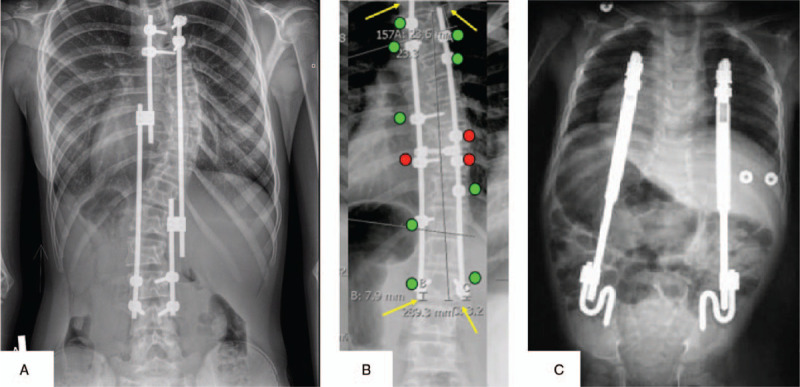
The example of radiogram patient with investigated system: (**A**) TGR (*traditional growing rod*), (**B**) GGS (*guided growth system*), (**C**) VEPTR (*verticale expandible prosthetic titanium rib*).

The study was approved by the Bioethics Committee of the Medical University of Lublin (consent no. KE-0254/105/2015). Written informed consent was obtained from all study participants and their parents. The study involved 71 patients (53 girls and 18 boys) treated for spinal deformity at the Pediatric Orthopedic Clinic of the Medical University of Lublin in 2015–2016. The average age of the patients was 13.5 ± 3.54 years and the average time of treatment was 2 ± 2.8 years. The patients were divided into four groups of patients treated with the TGR, GGS, and VEPTR (Figure 1A–C). The fourth group comprised patients who required a revision procedure because of rod breakage (A). The systems were based on titanium, aluminum, and vanadium alloy containing approximately 90% of titanium.

Two-rod constructions were used in all systems. Additionally, transpedicular screws with two domino connectors were used in the TGR system. In GGS, in addition to the screws, one transverse connector was used on the apex of curvature. Rib and pelvic or laminar hooks were used as an anchor points in the VEPTR group. In the study groups, tissue samples were taken during the lengthening procedure. The GGS group was sampled during the last operation performed with the spinal fusion. Samples taken from the patients before starting the treatment were used as control. Detailed patient characteristics are shown in Table 1.

**TABLE 1:** Demographic Data of Recruited Patients (n = 71)

	TGR (n = 38)	VEPTR (n = 9)	GGS (n = 10)	A^∗^ (n = 14)
Sex (male/female)	8/30	4/5	0/10	6/8
Age, y, mean ± SD	13.5 ± 3.8	12.3 ± 1.6	13.4 ± 1.6	12.6 ± 2.7
BMI	16.5 ± 4.3	17.5 ± 1.85	21.4 ± 10	16 ± 4.2
No. of segments	14 ± 2	16.9 ± 5	11 ± 3.2	13.5 ± 2.6
No. of anchors	7.9 ± 4.5	5 ± 2.5	11	7 ± 2.4

BMI indicates body mass index; GGS, guided growth systems; SD, standard deviation; TGR, traditional growing rod; VEPTR, vertical expandable prosthetic titanium rib.

∗ A—patients who required a revision procedure because of rod breakage.

### Sample Collection

**Figure 2:**
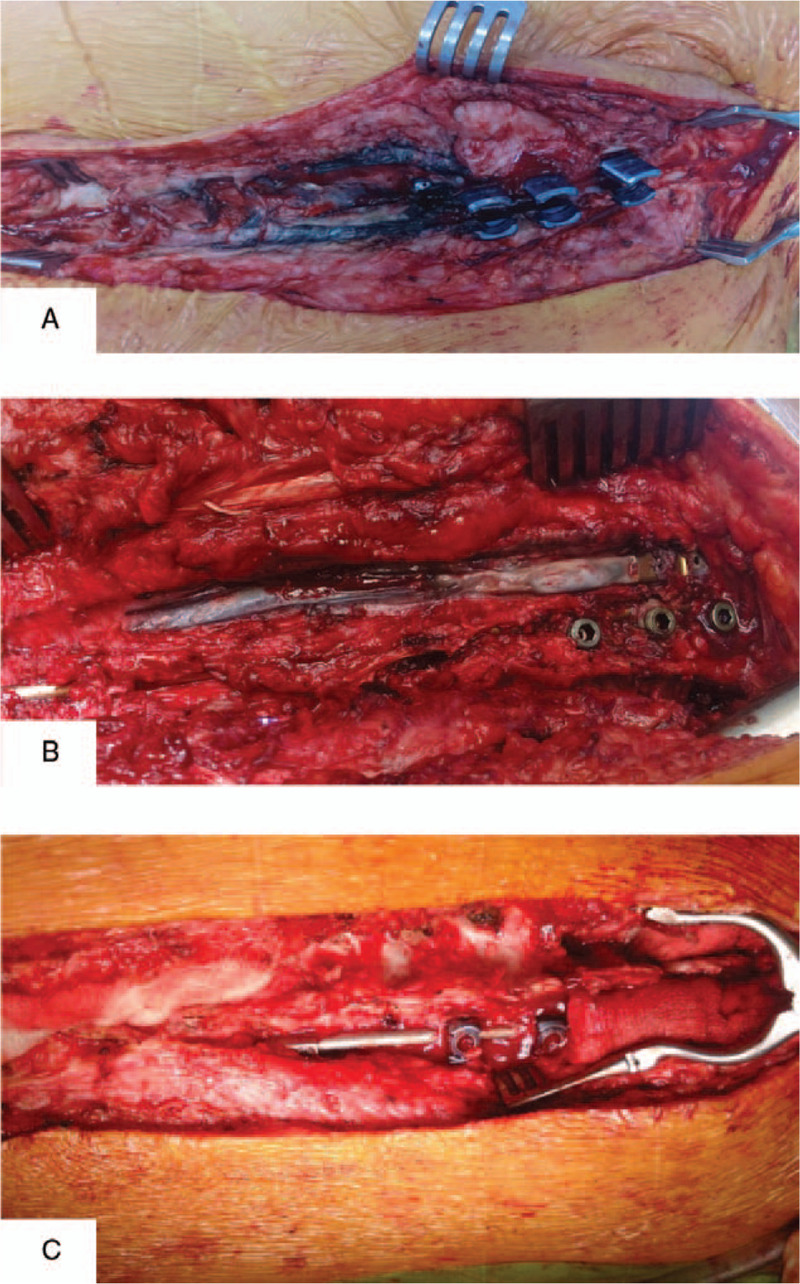
The fragment of subcutaneous tissue and contaminated tissue located near the implants obtained for investigated systems (intraoperative image): (**A**) GGS, (**B**) VEPTR, (**C**) TGR.

Peripheral blood samples (3 mL) were collected into a sterile test polypropylene tube with ethylenediaminetetraacetic acid containing no trace elements (S-Monovette, SARSTEDT AG & Co. KG), frozen in liquid nitrogen, and stored at −80°C until analysis. Samples of hair and nails were washed with distilled water, defatted with acetone, and washed with ethylenediaminetetraacetic acid to remove adsorbed ion metals. They were stored in a polypropylene tube. During the surgery, fragments of subcutaneous tissue (ST) located ca. 0.5 cm under the skin incision area and contaminated tissue with visible metallic debris located near the implants were collected (Figure 2A–C), frozen in liquid nitrogen, and stored at −80°C until analysis.

### Sample Preparation

The samples were accurately weighted, quantitatively transferred into CX-100 Teflon vessels, and mineralized in TOPWave apparatus (Analytik Jena, Germany) with an oxidative mixture composed of 69% nitric acid (Suprapure Merck Darmstadt, Germany) and deionized water (Ultrapure Millipore Direct-Q-R 3UV Merck) in a ratio of 2:1 (v/v).^11^ The conditions of the process are presented in Table 2. The supernatant was taken for ICP-OES analysis and solid residues were assessed based on microscopic analysis.

**TABLE 2:** Conditions of the Mineralization Procedure

Step	Temperature, °C1	Pressure, bar	Ramp, min	Time, min	Magnetron Power (%)
1	160	50	5	5	90
2	190	50	1	10	90
3	50	0	1	10	0
4	50	0	1	10	0
5	50	0	1	1	0

### Microscopic Analysis

The particles were examined with the use of an Olympus BX41 light microscope (Olympus, Hamburg, Germany). Images were registered with a photographic camera (Olympus Camedia C-5060, Hamburg, Germany). Images obtained at 40× magnification were binarized and segmented (Fiji image processing). ImageJ software was used to measure particle size parameters.^12^ Feret diameter and circularity were selected as the most representative shape descriptors. The results were analyzed in GrapPad Prism 5.0 (GrapPad Software, San Diego, CA). The surface layer of the samples was observed with a TESCAN VEGA3 LMU scanning electron microscope (SEM) (Tescan, Brno, Czech Republic). A graphite double-sided adhesive tape was covered with the sample; afterwards, the sample was dehydrated and sputter-coated with gold (Emitech K550X, Quorum Technologies, UK). The analyses were performed at acceleration voltage of 30 kV.

### ICP-OES Analysis

**Figure 3:**
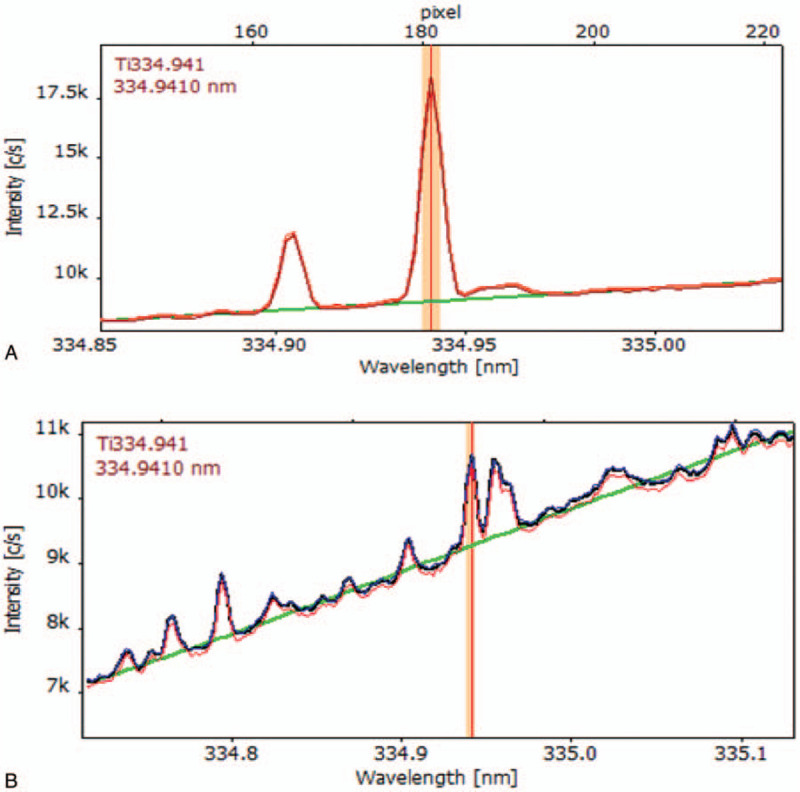
Example of titanium signal recorded for mineralized blood (**A**) and paraspinal tissue (**B**).

The analysis of the main components of the titanium alloy was performed using inductively coupled plasma optical emission spectrometry (ICP-OES) (PlasmaQuant PQ 9000, Analytik Jena AG, Germany). Aluminum and vanadium were not found in the investigated samples (their content was below the limit of detection). The working parameters of the apparatus and the calibration parameters of titanium are summarized in Tables 3 and 4, respectively. An example of a titanium signal recorded for mineralized paraspinal tissue and blood is presented in Figure 3A and B. The procedure was validated based on Human Hair Certified Reference Material (WSC, China), Human Whole Blood Certified Reference Material (Sero AS, Norway), and Human Plasma Control for trace elements (Recipe, Germany).

**TABLE 3:** Working Parameters of ICP-OES

Analytical line, nm	334.91
Plasma power, W	1300
Plasma gas flow, L/min	12
Auxiliary gas flow, L/min	0.5
Nebulizer gas flow, L/min	0.6
Plasma monitoring mode	axial
Peak evaluation (pixels)	3

ICP=OES indicates inductively coupled plasma optical emission spectrometry.

**TABLE 4:** Calibration Curve Parameters

Calibration curve equation	Ints. = 2353.2780 × C_Ti_ (μg/L) + 22.0818
Blank detection limit, μg/L	0.03668
Blank quantification limit, μg/L	0.11004
Experimental detection limit, μg/L	0.25
Recovery for 0.25 μg/L standard solution (%)	101.6

### Statistical Analysis

Statistical analysis was carried out with the use of Statistica 13.0 program (StatSoft, Poland). The following parameters were assessed: arithmetic mean, median, minimum and maximum values of variables, and standard deviation (SD). Comparison between the investigated groups was made using a Kruskal-Wallis multiple comparison test. Correlations were analyzed based on Spearman rank test.

## RESULTS

### The Level of Titanium in Subcutaneous and Paraspinal Tissue

**Figure 4:**
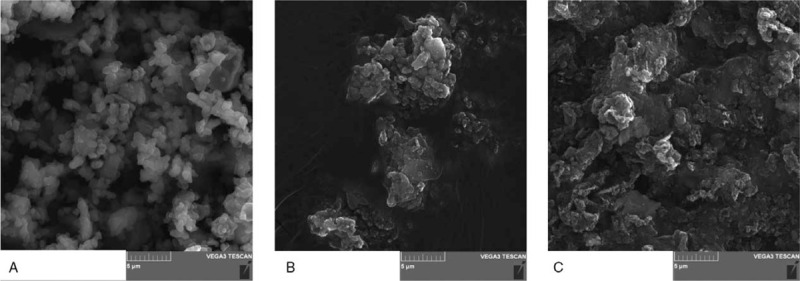
Scanning electron microscopy images of particles present in the solution after mineralization of paraspinal tissue from a patient with (**A**) GGS, (**B**) TGR and (**C**) VEPTR systems.

**Figure 5:**
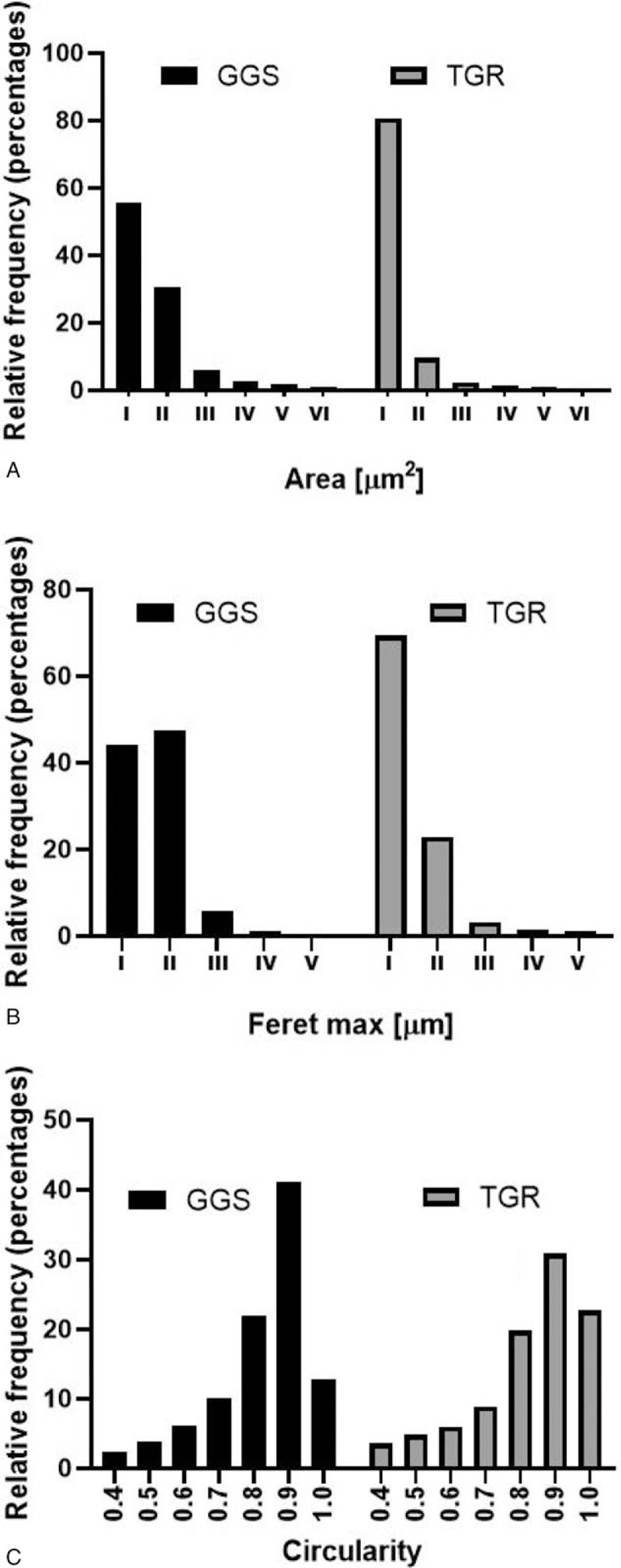
Analysis of particle area (**A**), dimension (Faret max) (**B**), and circularity (**C**) obtained with the use of Fiji image processing. (a) I ≤ 10μm^2^; II ≤ 20μm^2^; III ≤ 30μm2;IV ≤ 40μm^2^; V ≤ 50μm^2^; VI ≤ 60μm^2^; (b) I ≤ 5μm; II ≤ 10μm; III ≤ 15μm; IV ≤ 20μm; V ≤ 25μm.

**Figure 6:**
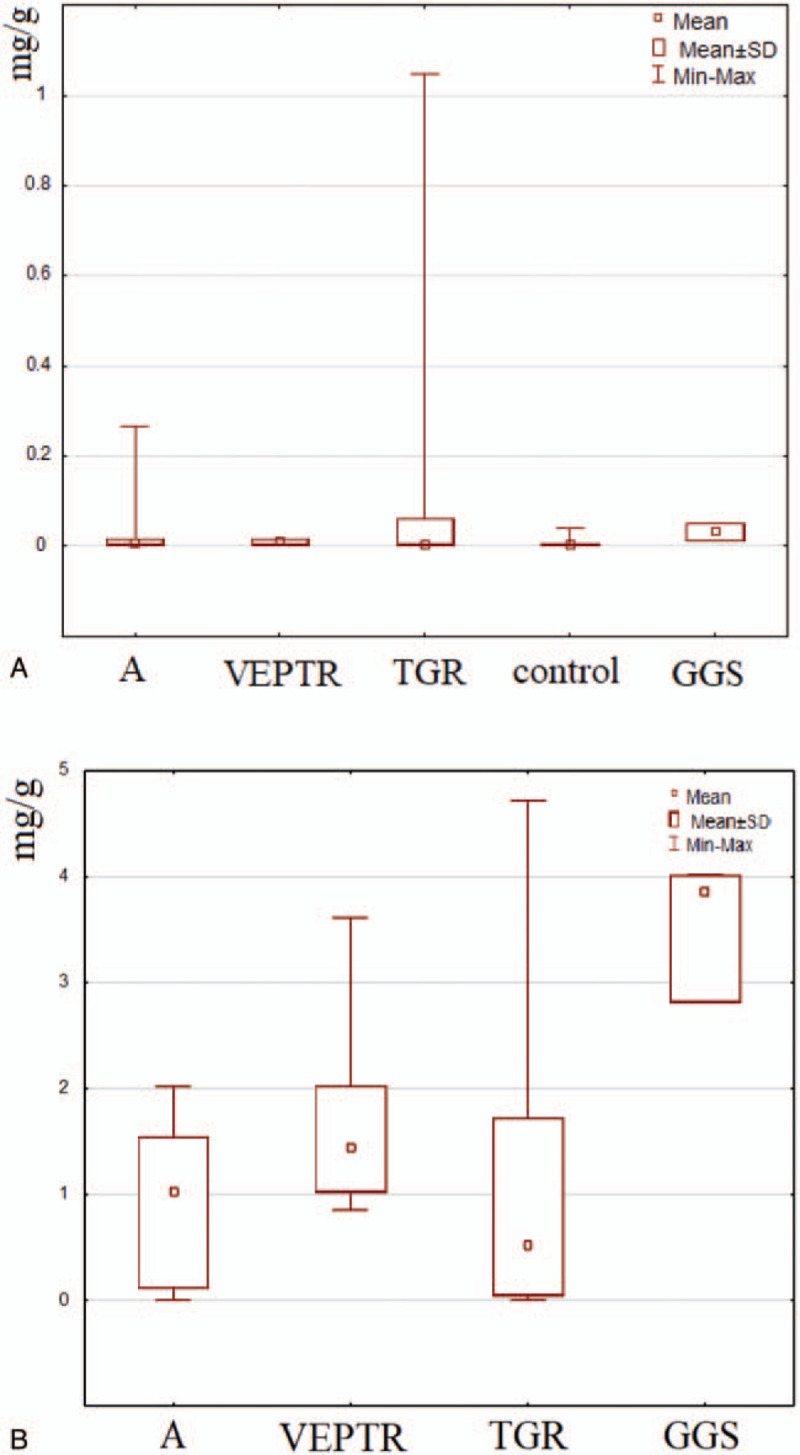
Results of titanium analysis for the subcutaneous (**A**) and paraspinal tissue (**B**). A - patients who required a revision procedure.

Initially, the presence of titanium in subcutaneous and paraspinal tissue was assessed based on observation and microscopic visualization. No metallic debris was visible in patients with the TGR system. In turn, metallic staining of paraspinal tissue was observed in the case of the GGS and VEPTR systems (Figure 2A–C). In the case of VEPTR, metallosis was visible along the entire length of the stabilization; in turn, in GGS, metallic debris was found mainly in the proximal thoracic and distal lumbar region due to the presence of structural elements moving relative to each other. The detailed microscopic observation of tissue mineralizates revealed the presence of small particles of metals also in the paraspinal tissue from the TGR patients (Figure 4A–C). The analysis of the particle dimension and area with the use of Fiji image processing showed that ca. 90% of particles in the paraspinal tissue were up to 10 μm in diameter, and the largest amount of small particles (<5 μm) was observed in the TGR system (Figure 5A–C). Moreover, the calculated circularity values indicated a round shape of a majority of the particles (the values obtained were >0.8, whereas the value of 1.0 indicates a perfect circle). Some particles had a size in a nm range and, therefore, they are not shown in the histograms. To estimate the content of soluble forms of titanium in the subcutaneous and paraspinal tissue, quantitative analysis was carried out with the use of ICP-OES. The results are presented in Figure 6A and B. As can be seen, the average amount of titanium in the ST was low. No statistically significant differences between the investigated groups of patients were found and the level of titanium was comparable to the control. In turn, the average titanium content in the paraspinal tissue in patients with the GGS systems differed significantly (*P* < 0.05) from that in patients with VEPTR and TGR and was two- to three-fold higher than the average value found in the other investigated groups. The relationships between the titanium level and sex, BMI, number of segments, anchors, and rods were assessed based on Spearman rank test. The high value of the correlation coefficient in Spearman test (0.8117) showed that the level of titanium increased with an increased BMI in the case of the VEPTR system, whereas no correlation was found for the other systems and the group subjected to the revision procedure. No statistically significant correlation (*P* < 0.05) was observed between the titanium level and the sex of the patients as well as the number of segments, anchors, and rods (Table S1, S2).

### The Level of Titanium in Blood

**Figure 7:**
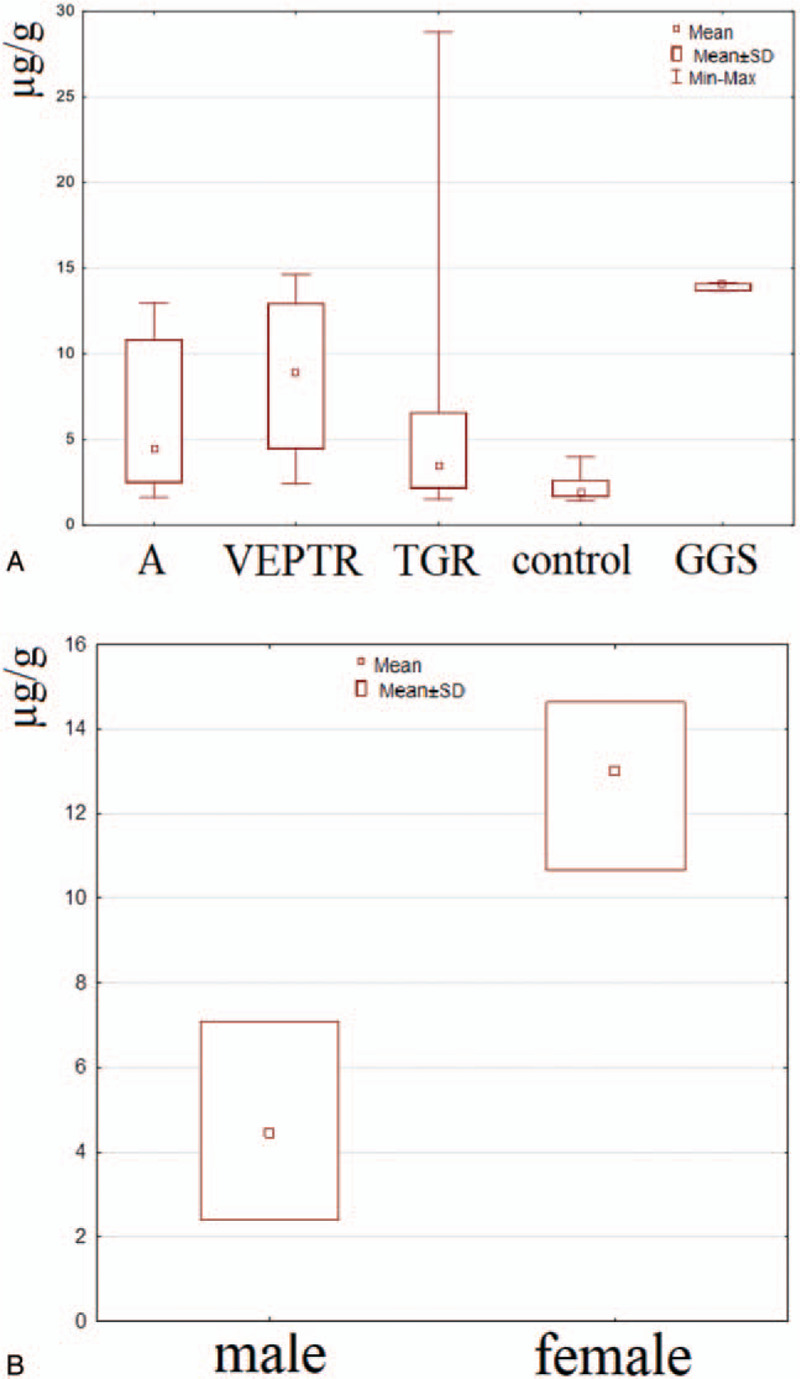
Titanium level in blood samples from patients with the investigated system (**A**) and in male and female patients with VEPTR system (**B**). A - patients who required a revision procedure.

Small particles of metal can be easily transformed to the soluble form and then migrate to the cardiovascular system; therefore, the content of titanium in blood was analyzed in the next step of our investigation. As can be seen in Figure 7A, an increased level of titanium compared with the control was noted in all the groups of patients. Moreover, similar to the results obtained for the ST, the highest content of titanium in blood was observed in patients with the GGS system. No statistically significant difference was noted between the groups of patients with VEPTR and TGR, and the determined level of Ti was significantly lower than in the case of GGS. No statistically significant correlation between the BMI and the titanium blood level was observed. However, taking into consideration the sex of the patients, a positive correlation was observed in the group with the VEPTR system (Figure 7B, Table S1). No statistically significant correlation between the titanium level and the number of segments, anchors, and rods was noted. The detailed data are shown in Table S3.

### The Level of Titanium in Hair and Nails

The soluble forms of heavy metals can migrate with blood to peripheral tissue; therefore, the analysis of their level in nails and hair may be helpful for assessment of the risk of accumulation. However, in our investigations, no titanium was detected in the nails and hair of the studied patients (the limit of detection for ICP-OES determination of titanium was ca. 0.04 μg/L).

## DISCUSSION

The term EOS describes a heterogeneous group of potentially life-threatening spinal deformities. Some of them may improve spontaneously, whereas surgical intervention is required in other cases. The treatment of EOS is a very difficult and challenging process. It is intended to prevent the development of deformities and to facilitate further growth of the spine and chest, resulting in normal development of the lungs and respiratory efficiency.^13–15^ The development of surgical techniques and their common use imposes increasing requirements on the quality of materials applied in medical implants. High mechanical strength, biocompatibility, and resistance to corrosive destruction in the tissue environment are expected. With its properties, titanium is widely used as an alloy with 6% of aluminum and 4% of vanadium (Ti-6Al-4 V) for production of orthopedic implants. In addition to the obvious benefits, surgical treatment of spinal defects carries the risk of a long-term presence of implants in the organism, which may result in release of metal ions into surrounding tissues and blood. Released metal particles can contribute to allergic and inflammatory reactions as well as carcinogenesis. The present study revealed the presence of metallic debris and increased content of soluble forms of titanium in paraspinal tissue, especially in the GGS systems. This observation is in agreement with literature reports. Morell and McCarthy^16^ observed local metallosis in patients with the steel Shilla system and the metallic debris was up to 45 μm in diameter. Macroscopically visible impurities were also observed in a caprine animal model.^17^ Morell and McCarthy and Singh *et al* also reported tissue inflammation with characteristics of metallosis.^16,18^ In the present study, we did not observe periimplantitis, and the metallic particles were much smaller (<25 μm), which may be related to the fact that the tested systems are based on a titanium alloy rather than a steel alloy. This suggests greater safety of titanium systems. As shown in the present study, the soluble forms of titanium migrated to the peri-implant tissues and the patients exhibited elevated levels of the metal, in comparison with the control. The presence of soluble titanium forms in peri-implant tissues has been observed by other researchers as well. Wang *et al*^9^ examined nine adult patients subjected to revision procedures accompanied by sampling tissue from different peri-implant areas. The detected titanium level ranged from 0.616 to 45.8 μg/g depending on the location. Similarly, we observed elevated blood titanium levels in all patients with titanium implants, which was in agreement with literature data; however, the level of titanium determined in blood was lower than that reported elsewhere.^19^ The results of determination of the titanium level in children with LSZ-4D sliding instrumentation after an average 6-year observation period indicated an approximately 2.8- to four-fold increase in the level of Ti ions in whole blood, compared to the control group, in which the Ti level was in the range of 30 to 40 μg/L.^19^ In our study, the initial titanium content was significantly lower and the difference can be explained by various environmental exposures to Ti. Cundy *et al*^20^ reported an approximately six-fold increase in the Ti ion content relative to the average preoperative level in 90% of children subjected to stabilization and spondylodesis based on titanium alloy implants. Richardson *et al*^10^ noted elevated serum levels of Ti ions in adults after instrumented spinal arthrodesis 26 months after the surgery. In turn, Ipach *et al*^13^ did not observe statistically significant changes 12 months after spinal fusion surgery in adults. They found no correlation with the number of stabilized segments or the length of the implants. Mechanical damage to the implant, which involves certain instability of the system and appearance of additional friction between the elements of the structure, may be expected to increase the release of ions. No such correlation was found in the present study and in investigations conducted by Kasai *et al*.^21^ The present study showed that, irrespective of the system used, metallic particles were released into the peri-implant tissue, and the greatest amounts of titanium particles were detected in patients with the GGS system. This is probably associated with the friction between implant components, whereas the components in the other systems are immobile relative to each other (slight metallosis may result from movement of the spine or surrounding tissues *vs.* the implant). Nevertheless, the concentration of soluble titanium forms in both ST and blood was only slightly higher than in the control and did not exceed the allowable levels. In our investigations, no titanium was detected in the nails and hair of the studied patients.

## CONCLUSION

Titanium-based biomaterials are used in surgical treatment of EOS; however, they remain in the body for several years and Ti can be released into surrounding tissues and migrate to blood. The study consisted in assessment of the level of titanium in EOS patients with different surgical systems, including TGR, GGS, and VEPTR.

Our investigation revealed an increased level of titanium in blood, compared with the control, in all groups of patients and the highest content was observed in patients with the GGS system; however, it did not exceed the allowable levels. Additionally, the titanium content in the paraspinal tissue in patients with the GGS systems was two- to three-fold higher than the average value found in the other investigated groups. This is probably a result of the increased friction between implant components, compared to the other surgical systems. No statistically significant correlation was observed between the titanium level in the blood and paraspinal tissue and the sex of the patients as well as the number of segments, anchors, and rods, except the VEPTR patients, where the level of titanium increased with an increased BMI.

**Key Points**

- Seventy-one patients (13.5 ± 3.54 years’ old) treated for spinal deformity.
- TGR, GGS, and VEPTR systems were used.
- Samples of paraspinal tissue, blood, nails, and hair were collected and titanium level was quantified.

## Supplementary Material

Supplemental Digital Content
