# Supplementary material for: Comparison of Different Surgical Systems for Treatment of Early-onset Scoliosis in the Context of Release of Titanium Ions
Source: Spine (Phila Pa 1976). 2020 Dec 7;46(10):E594–601. doi: 10.1097/BRS.0000000000003846 (PMC8614546; doi:10.1097/BRS.0000000000003846)
Supplement: Supplemental Digital Content [file brs-46-e594-s001.docx]

Tab. S1. Correlation coefficient (R) between content of titanium and investigated factors.

| Group | blood | | paraspinal | |
| --- | --- | --- | --- | --- |
|  | sex | BMI | sex | BMI |
| A* | 0.1134 | -0.3273 | -0.333 | -0.0501 |
| VEPTR | 0.9158 | 0.0286 | 0.333 | 0.8117 |
| TGR | 0.0121 | -0.2592 | -0.1978 | -0.2385 |
| control | -0.2591 | 0.0729 | ---- | --- |
| GGS | ** | -0.9965 | ** | -0.219 |

- A - patients who required a revision procedure because of rod breakage

** in GGS groups were only women

Tab. S2. Correlation coefficient (R) between content of titanium in tissue and investigated factors

| Group | No.  segments | No.  anchors | correlation coefficient (R) | |
| --- | --- | --- | --- | --- |
|  |  |  | No. anchors | No. segments |
| TGR | 14 ± 2 | 7.9 ±4.5 | 0.0143 | 0.0406 |
| VEPTR | 16.9 ± 5 | 5 ± 2.5 | -0.0883 | -0.676 |
| GGS | 11 | 11 | ** | ** |
| A* | 13.5 ± 2.6 | 7 ± 2.4 | -0.2334 | 0.2027 |

*A - patients who required a revision procedure because of rod breakage

** in GGS all patients have eleven anchors and eleven stabilized segments

Tab. S3. Correlation coefficient (R) between content of titanium in blood and investigated factors

| Group | No. segments | No.  anchors | correlation coefficient (R) | |
| --- | --- | --- | --- | --- |
|  |  |  | No. anchors | No. segments |
| TGR | 14 ± 2 | 7.9 ±4.5 | 0.0503 | 0.0565 |
| VEPTR | 16.9 ± 5 | 5 ± 2.5 | 0.1988 | 0.2143 |
| GGS | 11 | 11 | ** | ** |
| A* | 13.5 ± 2.6 | 7 ± 2.4 | 0.1330 | 0.3134 |

*A - patients who required a revision procedure because of rod breakage

** in GGS all patients have eleven anchors and eleven stabilized segments
